# An Ultrasound Radiomics Nomogram for Preoperative Prediction of Central Neck Lymph Node Metastasis in Papillary Thyroid Carcinoma

**DOI:** 10.3389/fonc.2020.01591

**Published:** 2020-09-04

**Authors:** Shi-Chong Zhou, Tong-Tong Liu, Jin Zhou, Yun-Xia Huang, Yi Guo, Jin-Hua Yu, Yuan-Yuan Wang, Cai Chang

**Affiliations:** ^1^Department of Ultrasonography, Fudan University Shanghai Cancer Center, Shanghai, China; ^2^Department of Oncology, Shanghai Medical College, Fudan University, Shanghai, China; ^3^Department of Electronic Engineering, Fudan University, Shanghai, China; ^4^Key Laboratory of Medical Imaging, Computing and Computer-Assisted Intervention, Shanghai, China

**Keywords:** ultrasound, papillary thyroid carcinoma, lymph node, metastasis, radiomics

## Abstract

**Purpose:** This study aimed to establish and validate an ultrasound radiomics nomogram for the preoperative prediction of central lymph node (LN) metastasis in patients with papillary thyroid carcinoma (PTC).

**Patients and Methods:** The prediction model was developed in 609 patients with clinicopathologically confirmed unifocal PTC who received ultrasonography between Jan 2018 and June 2018. Radiomic features were extracted after the ultrasonography of PTC. Lasso regression model was used for data dimensionality reduction, feature selection, and radiomics signature building. The predicting model was established based on the multivariable logistic regression analysis in which the radiomics signature, ultrasonography-reported LN status, and independent clinicopathologic risk factors were incorporated, and finally a radiomics nomogram was established. The performance of the nomogram was assessed with respect to the discrimination and consistence. An independent validation was performed in 326 consecutive patients from July 2018 to Sep 2018.

**Results:** The radiomics signature consisted of 23 selected features and was significantly associated with LN status in both primary and validation cohorts. The independent predictors in the radiomics nomogram included the radiomics signature, age, TG level, TPOAB level, and ultrasonography-reported LN status. The model showed good discrimination and consistence in both cohorts: C-index of 0.816 (95% CI, 0.808–0.824) in the primary cohort and 0.858 (95% CI, 0.849–0.867) in the validation cohort. The area under receiver operating curve was 0.858. In the validation cohort, the accuracy, sensitivity, specificity and AUC of this model were 0.812, 0.816, 0.810, and 0.858 (95% CI, 0.785–0.930), respectively. Decision curve analysis indicated the radiomics nomogram was clinically useful.

**Conclusion:** This study presents a convenient, clinically useful ultrasound radiomics nomogram that can be used for the pre-operative individualized prediction of central LN metastasis in patients with PTC.

## Introduction

The incidence of thyroid cancer has increased significantly in last two decades (1, 2), and the papillary thyroid carcinoma (PTC) accounts for the majority of thyroid cancers (3, 4). In the newly diagnosed thyroid cancers, the proportion of papillary thyroid microcarcinoma (PTMC), defined as PTC tumor ≤ 1 cm in diameter, increases dramatically (5–7). Several studies have reported that PTMC progresses slowly and follow up may be preferred over surgical treatment, and lymph node (LN) metastasis has been regarded an indication to surgical treatment for PTMC (8, 9). Moreover, the judgement of LN metastasis not only affects the staging of PTC, but also influences its treatment and the extent of resection (10–12). High frequency ultrasound (US) can be employed to diagnose lateral cervical LN metastasis accurately and guide the biopsy (12–14). However, the accuracy of US is relatively low in the diagnosis of central cervical LN metastasis due to the overlying thyroid gland (15, 16). The accuracy is only about 70%, even combined with computer tomography (CT) (17, 18).

There is evidence showing that some gray scale features of US has a close relationship with neck LN metastasis of PTC (19–21). However, the diagnosis varies greatly among different US physicians due to the considerable subjectivity of the understanding and application of diagnostic criteria. Radiomics, based on machine-learning, emerging in recent years, is a method that extracts a large amount of features from the radiographic medical images using data-characterization algorithms, which is helpful for the interpretation of tumor features (22). It can not only quantitatively extract and analyze the features of US images, but also identify the tumor information from images that can't be macroscopically recognized (23, 24). Available studies have revealed that radiomics can be used to predict the cervical LN metastasis in PTC patients (25). In our previous study, the PTC ultrasound images were extracted with radiomics method and then used for the prediction of cervical LN metastasis in the PTC patients (26). However, their performance were not good enough, which might be ascribed to the small sample and the prediction based on the image information only extracted relying on radiomics. To date, no study with large sample size has been conducted to investigate the prediction of central LN metastasis in PTC by US radiomics except for ours.

The present study aimed to establish a prediction model for central LN metastasis in a relatively large scale population of PTC patients based one the US radiomics, biochemical results and US findings, further validate this model in clinical cases, and evaluate its clinical significance.

## Patients and Methods

### Patients and Clinical Characters

This retrospective study was approved by the Ethics Committee of Cancer Hospital of Fudan University and complied with the Helsinki Declaration. The informed consent requirement was waived. The data in this study were obtained from a database of patients who received surgical treatment of thyroid lesions in our hospital. The inclusion and exclusion criteria were as follows.

### Inclusion Criteria

(1) According to the American College of Radiology (ACR) Thyroid Imaging Reporting and Data System (TI-RADS) (27), ultrasonography showed category IV (Suspicious for Malignancy) or V (Malignant), and then fine needle aspiration (FNA) was performed. Cytological examination showed categories V (Suspicious for Malignancy) or VI (Malignant) according to the Bethesda system (28). They received surgical treatment in our hospital; (2) Patients received prophylactic central neck dissection in the surgery; (3) Patients received initial surgery; (4) Unifocal PTC was pathologically diagnosed; (5) The clinical information (including thyroid hormones) was complete; (6) The pre-operative images met the requirements, and ultrasonography showed the results about the central cervical LN and thyroid lesions.

### Requirements for US Images

(1) Images showed as many malignant features as possible on the axial plane; (2) Images displayed the relationship between the lesion and the thyroid capsule; (3) Images showed the longest diameter of the lesion; (4) Images had no distance, area, elasticity and Doppler measurements; (5) Image acquisition and US diagnosis were done with the ultrasound device Aixplorer [Supersonic Imagine] by several US physicians with more than 10 years' experience in the thyroid ultrasonography.

### Exclusion Criteria

(1) Patients received preoperative interventional therapies (such as radiofrequency and microwave therapies) or head and neck radiotherapy; (2) Postoperative pathological examination showed concomitant non-PTC components in the lesion (such as atypical hyperplasia, follicular tumors, medullary carcinomas, undifferentiated carcinomas and metastatic carcinomas, etc.); (3) postoperative pathological examination showed multifocal PTC; (4) the clinical information was incomplete; (5) the US images didn't meet the requirements.

The primary cohort included 609 patients (176 males and 431 females) with the mean age of 42.07 ± 11.49 years (range: 22–82 years) from 2,219 consecutive patients who received surgical treatment between January 2018 and June 2018. In addition, 326 patients (99 males and 227 females) were included in the validation cohort with the mean age of 43.48 ± 11.81 years (range: 18–74 years) from 1,311 consecutive patients who received surgical intervention between July 2018 to September 2018. The level of attrition in this study was consistent with previously reported (29).

Baseline clinical characteristics, including gender, age, thyroid stimulating hormone (TSH), thyroglobulin (TG), thyroglobulin antibodies (TGAB), and thyroid peroxidase antibody (TPOAB), and cytological findings after FNA were collected from medical records. According to the TI-RADS criteria of ACR (27), the US images of each lesion were classified and scored before surgery. The cervical LN metastasis was diagnosed by ultrasonography before surgery, and it was recorded concomitantly with US-reported LN status from US report system. TSH, TG, TGAB and TPOAB were detected within 1 week before surgery. According to the clinical experience, the thresholds for TSH, TG, TGAB, and TPOAB were as follows: TSH, ≥4.94 ng/ml; TG, ≥77 ng/ml; TGAB, ≥4.11 IU/ml; TPOAB, ≥5.61 IU/ml. The cytological examination after FNA showed category V or VI according to the Bethesda system. The TR score of lesions on US images corresponded to the TI-RADS category: TI-RADS 4 ≥4 points, TI-RADS 5 ≥ 7 points.

The demographics were compared between LN metastasis positive and negative groups in both primary and validation cohorts; independent sample *t*-test was used to assess the difference in the age and TR score between two groups in both primary and validation cohorts; Chi-square test was employed to evaluate the differences in the gender, TSH, TG, TPOAB, TGAB, Bethesda category, and US-report LN status between two groups in both primary and validation cohorts. The proportion of LN metastasis positive patients was compared with Chi-square test between primary and validation cohorts.

### Surgery and Pathology

Patients received lobectomy and isthmectomy or total thyroidectomy depending on the clinical TNM stage (12). All the patients underwent prophylactic central neck dissection. For patients with lateral cervical LN metastasis, lateral cervical LN dissection was done. The resected thyroid tissues were processed for pathological examination (including the determination of unifocal or multifocal PTC). The resected LNs were also subjected to pathological examination, and LN metastasis was determined.

### US Images and US Radiomics Signatures

All the patients underwent preoperative US examination of the thyroid and central cervical LN with an US machine [Supersonic (Acoustic)]. The parameters were consistent among patients: image depth, 3 cm; gain, 53%; focus parallel to the lesion. The images of thyroid lesion were stored with DICOM format. US physicians with more than 10 years' experience in the thyroid ultrasonography were responsible for the pre-operative acquisition of US images, TI-RADS classification and assessment of cervical LN status. Preoperative US findings on LN metastasis were used as the US-reported LN status. Positive LN metastasis on US was defined as US findings suggestive of LN metastasis, and negative LN metastasis on US was defined as US findings suggestive of “undetectable lymph nodes,” “inflammatory lymph nodes,” and “lymph nodes” in the absence of metastasis.

An axial grayscale US image meeting the requirements was selected from each patient, and the lesion was delineated for radiomics analysis. This was done by a clinician with more than 10 years' experience in thyroid ultrasonography (US doctor-1). According to the ACR, American Thyroid Association (ATA) and American Association of Clinical Endocrinologists (AACE) guidelines, the US image features of PTC were defined (12, 27, 30). Ten parameters were included: demographic information and tumor size, shape, orientation, position, margin, boundary, echo pattern, posterior acoustic pattern and calcification. Then, the software “PTC cervical LN metastasis prediction system” developed by the Department of Electronic Engineering, Fudan University was used to input DICOM images after delineation, followed by extraction of image features. A 4-step feature selection method was employed to select the most effective radiomics features. First, a 2-sided Wisconsin rank sum test was used to select features related with central cervical LN status. Then, a geneti c algorithm combing with minimum-redundancy-maximum-relevance was applied to remove the redundant features. A sparse representation classification was used to sequence the remaining features according to their importance. The top 50 important features were selected. Finally, the optimal features were sorted from these features with the least absolute shrinkage and selection operator (LASSO) for the establishment of a formula of US radiomics features after dimensionality reduction. The detailed methods used were published in our previous study (26, 31). The linear combination of each selected feature was done according to their weighted coefficients, and a weighted formula was established to calculate the score of US radiomics signature for each patient. The Mann-Whitney U test/independent samples *t*-test was used to assess the association between US radiomics signature and LN metastasis in validation cohort after stratification (age, gender, serum indicators, and US findings). All radiomics feature extraction and selection methods were performed in MATLAB R2015b (Mathworks, Inc.).

The reproducibility of US radiomics features extraction was evaluated based on the intra-operator and inter-operator findings. 2 weeks after extraction of US radiomics features in the primary cohort (US doctor-1 first), the same US physician extracted the US radiomics features with the same procedure for the evaluation intra-operator agreement on features extraction (US doctor-1 s). Another clinician with more than 2 years' experience in the thyroid ultrasonography (US doctor-2) performed the same examination in the primary cohort for the evaluation inter-operator agreement on features extraction by comparing findings between two physicians. An independent samples *t*-test was used to evaluate the intra- and inert-operator differences. The inter- and intra-class correlation coefficients (ICCs) >0.75 were suggestive of good agreement.

In addition, 50 consecutive patients with PTC meeting the inclusion criteria were included as the control cohort, aiming to confirm the stability of US radiomics features collected from primary cohort, and ultrasound examination was done with Voluson E8 [GE]. The parameters and processes used for image acquisition and analysis were the same to those in primary cohort and validation cohort. According to the formula established based on the US radiomics features from primary cohort, the US radiomics features were extracted from both validation cohort and control cohort, and then the Receiver Operating Characteristic (ROC) of US radiomics features in predicting cervical central LN metastasis was delineated. Z test was used for the comparison of Area Under the Curve (AUC) between them.

### Prediction Model and Clinical Significance

In the primary cohort, the multivariable logistic regression analysis was performed based on the clinical predictors (age; gender; TSH; TG; TPOAB; TGAB; FNA Bethesda category; ACR score of PTC lesion; US-reported LN status) and US radiomics signature. Forward step-wise selection was applied by using the likelihood ratio test (32). The US radiomics nomogram, a two-dimensional image used to calculate the risk for a disease by quantifying each related risk factor, was established by using the selected predictors from multivariable logistic regression analysis. The calibration curve of US radiomics nomogram was delineated, and the Hosmer-Lemeshow test was used to evaluate the fitting of this curve (33). Harrell's C-index was determined to evaluate the discrimination performance of US radiomics nomogram. In the validation cohort, the calibration and discrimination performances were evaluated by calibration curve and C-index, respectively.

The diagnostic accuracy, sensitivity and specificity of the nomogram were determined in both primary and validation cohort. In the validation cohort, the ROCs of prediction model and pre-operative US diagnosis, AUCs were calculated, and Z test was employed for comparison. To determine the clinical significance of US radiomics nomogram, decision curve analysis was employed to quantify the net benefits at different threshold probabilities in validation cohort (34). The clinical impact curve was also plotted to investigate the ratio of false positive value to true positive value at different threshold risks.

### Statistical Analysis

Statistical analysis was carried out with R software (version 3.5.3 http://www.r-project.org). The package used in this study included “hmisc,” “grid,” “lattice,” “formula,” “ggplot2,” “rms,” “proc,” and “survival.” A value of two-sided *P* < 0.05 was considered statistically significant.

## Results

### Clinical Characteristics

The clinical characteristics of patients in both primary cohort and validation cohort are shown in Table 1. There was no marked difference in the LN metastasis between two cohorts. The proportion of patients positive for LN metastasis in primary cohort and validation cohort was 29.7 and 34.6%, respectively (*P* = 0.134). In addition, significant differences in some other clinical characteristics were also not observed in both the LN-positive group (*P* = 0.894 for age, 0.972 for gender, 0.258 for TSH, 0.831 for TG, 0.297 for TGAB, 0.068 for TPOAB, 0.602 for Bethesda category, 0.227 for TR score, and 0.349 for US-report LN status) and the LN-negative group (*P* = 0.541 for age, 0.449 for gender, 0.465 for TSH, 0.638 for TG, 0.906 for TGAB, 0.328 for TPOAB, 0.355 for Bethesda category, 0.182 for TR score, and 0.525 for LN-report LN status) between primary cohort and validation cohort. This suggests that the primary cohort and validation cohort were comparable in these clinical characteristics.

**Table 1:** Clinical characteristics of patients in primary cohort and validation cohort.

**Characteristic**	**Primary cohort**			**Validation cohort**		
	**LN+**	**LN–**	***P***	**LN+**	**LN–**	***P***
Age, mean ± SD, years	42.41 ± 11.79	42.49 ± 11.89	0.000^*^	43.46 ± 11.85	43.48 ± 11.77	0.000^*^
Gender, No. (%)			0.080^*^			0.020^*^
Male	72 (39.6)	106 (24.8)		45 (39.8)	54 (25.4)	
Female	110 (60.4)	321 (75.2)		68 (60.2)	159 (74.6)	
TSH, No. (%)			0.221			0.121
Normal (<4.94 ng/ml)	8 (4.4)	31 (7.3)		6 (5.3)	17 (8.0)	
Abnormal (≥4.94 ng/ml)	174 (95.6)	396 (92.7)		107 (94.7)	196 (92.0)	
TG, No. (%)			0.020^*^			0.007^*^
Normal (<77 ng/ml)	94 (51.6)	285 (66.7)		58 (51.3)	144 (67.6)	
Abnormal (≥77 ng/ml)	88 (48.4)	142 (33.3)		55 (48.7)	69 (32.4)	
TGAB, No. (%)			0.662			0.512
Normal (<4.11 IU/ml)	119 (65.4)	276 (64.6)		72 (63.7)	138 (64.8)	
Abnormal (≥4.11 IU/ml)	63 (34.6)	151 (35.4)		41 (36.3)	75 (35.2)	
TPOAB, No. (%)			0.592			0.463
Normal (<5.61 IU/ml)	123 (67.6)	304 (71.2)		78 (69.0)	150 (70.4)	
Abnormal (≥5.61 IU/ml)	59 (32.4)	123 (28.8)		35 (31.0)	63 (29.6)	
Bethesda category, No. (%)						
V	79 (43.4)	176 (41.2)	0.572	48 (42.5)	83 (39.0)	0.447
VI	103 (56.6)	251 (58.8)		65 (57.5)	130 (61.0)	
TR score, mean ± SD	7.84 ± 1.64	7.55 ± 1.61	0.166	7.93 ± 1.55	7.61 ± 1.63	0.130
US reported LN status, No. (%)			0.000^*^			0.000^*^
LN-positive	30 (16.5)	31 (7.3)		16 (14.2)	16 (7.5)	
LN-negtive	152 (83.5)	396 (92.7)		97 (85.8)	197 (92.5)	
Radiomics signature (interquartile range)	−0.223 (−0.097 to −0.337)	−0.424 (−0.313 to −0.550)	0.000^*^	−0.228 (−0.066 to −0.334)	−0.444 (−0.278 to −0.605)	0.000^*^

*P-value is derived from the univariable association between each clinicopathologic variables and LN status. Radiomics signature is reported as median and interquartile rang (IQR)*.

* *P < 0.05*.

### US Radiomics Signature

Finally, 23 effective US radiomics features were obtained (ratio: 26:1). A weighted formula was established based on these 23 features to calculate the score of US radiomics signature (Table 2). The C-index of US radiomics signature in the primary and validation cohorts was 0.793 (95% CI, 0.787–0.799) and 0.824 (95% CI, 0.815–0.833), respectively. In both primary cohort and validation cohort, significant difference was noted in the US radiomics signature between LN positive and LN negative patients. Subjects were stratified based on the clinical risk factors and then the features of US radiomics were compared between patients with and without cervical LN metastasis (Table 3).

**Table 2:** US radiomics feature and weighted coefficient after LASSO regression analysis.

**Lasso weighted coefficient**	**US radiomics feature in formula**
−0.05604	M Orientation
0.05054	histogram p Range
0.08764	cal std (roundness c)
−0.03682	glszmTextures-1.ZSV
0.03891	M spiculation-1
0.21772	M spiculation-2
0.07844	glszmTextures.GLN
0.03266	glszmTextures.LGZE
0.12425	histogram t MAD
−0.04177	histogram p entropy
0.08931	M overlapArea
−0.06091	glszmTextures−2.ZSV
0.09314	glcmTextures.maxpr
−0.01238	M Con sd p
−0.02044	M Diss sd p
0.03770	cal Area c max
−0.06361	post ngtdmTextures.Complexity
0.00582	cal sum (Perimeter c)
−0.02300	M Diss m t
−0.02095	M compactness
−0.05217	glrlmTextures.RLV
−0.05400	glszmTextures-3.ZSV
−0.00082	cal Area c min

**Table 3:** Relationship between US radiomics signature and LN metastasis in validation cohort after stratification.

		**US radiomics signature**		
	**Subgroup**	**LN metastasis (+)**	**LN metastasis (–)**	***P***
Gender	male	−0.225 (−0.354, −0.037)	−0.422 (−0.609, −0.226)	0.003
	female	−0.229 (−0.323, −0.075)	−0.448 (−0.604, −0.280)	0.000
US reported	LN+	−0.228 (−0.354, −0.121)	−0.437 (−0.585, −0.283)	0.000
	LN–	−0.228 (−0.328, −0.084)	−0.450 (−0.608, −0.302)	0.000
Bethesda category	V	−0.229 (−0.322, −0.092)	−0.404 (−0.572, −0.294)	0.000
	VI	−0.228 (−0.366, −0.129)	−0.442 (−0.602, −0.280)	0.003
TSH	Abnormal (≥4.94 ng/ml)	−0.192 (−0.327, −0.081)	−0.411 (−0.664, −0.283)	0.000
	normal (<4.94 ng/ml)	−0.228 (−0.334, −0.066)	−0.444 (−0.605, −0.279)	0.003
TG	Abnormal (≥77 ng/ml)	−0.216 (−0.328, −0.084)	−0.351 (−0.529, −0.202)	0.000
	normal (<77 ng/ml)	−0.459 (−0.610, −0.302)	−0.255 (−0.369, −0.111)	0.000
TGAB	Abnormal (≥4.11 IU/ml)	−0.162 (−0.239, −0. 134)	−0.400 (−0.612, −0.218)	0.000
	normal (<4.11 IU/ml)	−0.454 (−0.600, −0.309)	−0.264 (−0.350, −0.110)	0.000
TPOAB	Abnormal (≥5.61 IU/ml)	0.010 (−0.274, 0.148)	−0.315 (−0.512, −0.190)	0.005
	normal (<5.61 IU/ml)	−0.459 (−0.609, −0.304)	−0.242 (−0.340, −0.094)	0.000

*The score of US radiomics signature is expressed by median and quartile spacing*.

The intra- and inter-operator reproducibilities of US radiomics features extraction were further assessed. Results showed no significant difference neither between features extracted from the first and second time by the same US physician (*P* = 0.605), nor between features extracted by US doctor-1 and US doctor-2 (*P* = 0.738). The intra-class correlation coefficient of US doctor-1 in two extractions ranged from 0.845 to 0.962. The inter-class correlation coefficient of extraction by US doctor-1 and US doctor-2 ranged from 0.886 to 0.934. After validation of inter- and intra-operator reproducibilities, all outcomes were based on the features first extracted by US doctor-1.

**Figure 1:**
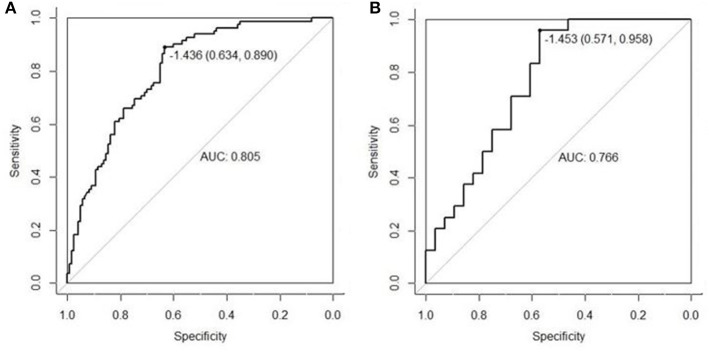
The radiomics features extracted from the primary cohort were applied to the validation cohort **(A)** and the control cohort **(B)**, and then ROC was delineated for the prediction of central cervical LN metastasis. Although the AUC in the validation cohort was higher than in the control cohort (0.805 vs. 0.766), Z test indicated no significant difference in diagnostic efficacy between two cohorts (*P* = 0.595). This suggests that the US radiomics features have same diagnostic performance across different US machines.

The AUC of US radiomics features was 0.805 (95% CI, 0.746–0.864) in the validation cohort and 0.766 (95% CI, 0.637 to 0.896) in the control cohort (Figures 1A,B). Z test showed no significant difference (*P* = 0.595).

### Prediction Model of US Radiomics Nomogram

**Figure 2:**
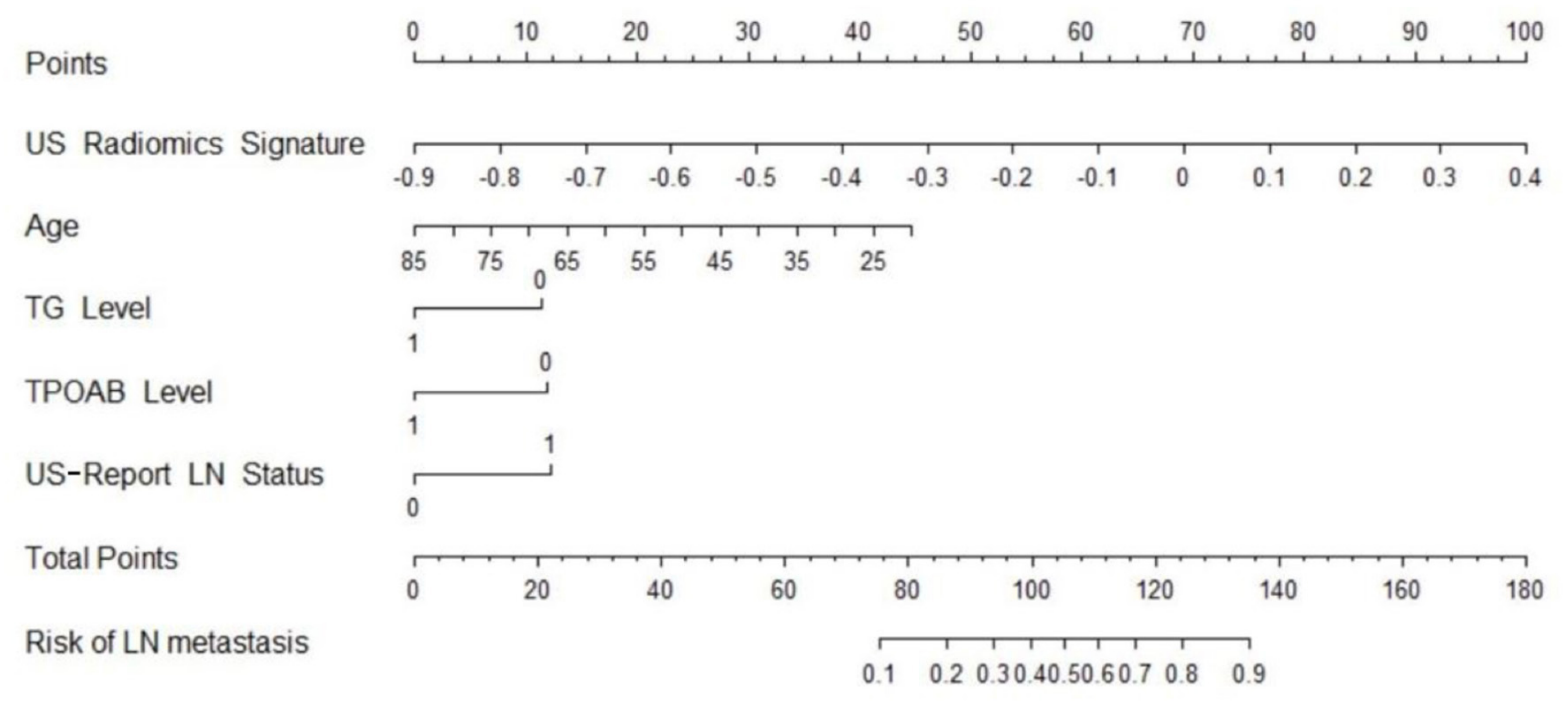
US radiomics nomogram used for prediction of central cervical LN metastasis in PTC patients.

**Figure 3:**
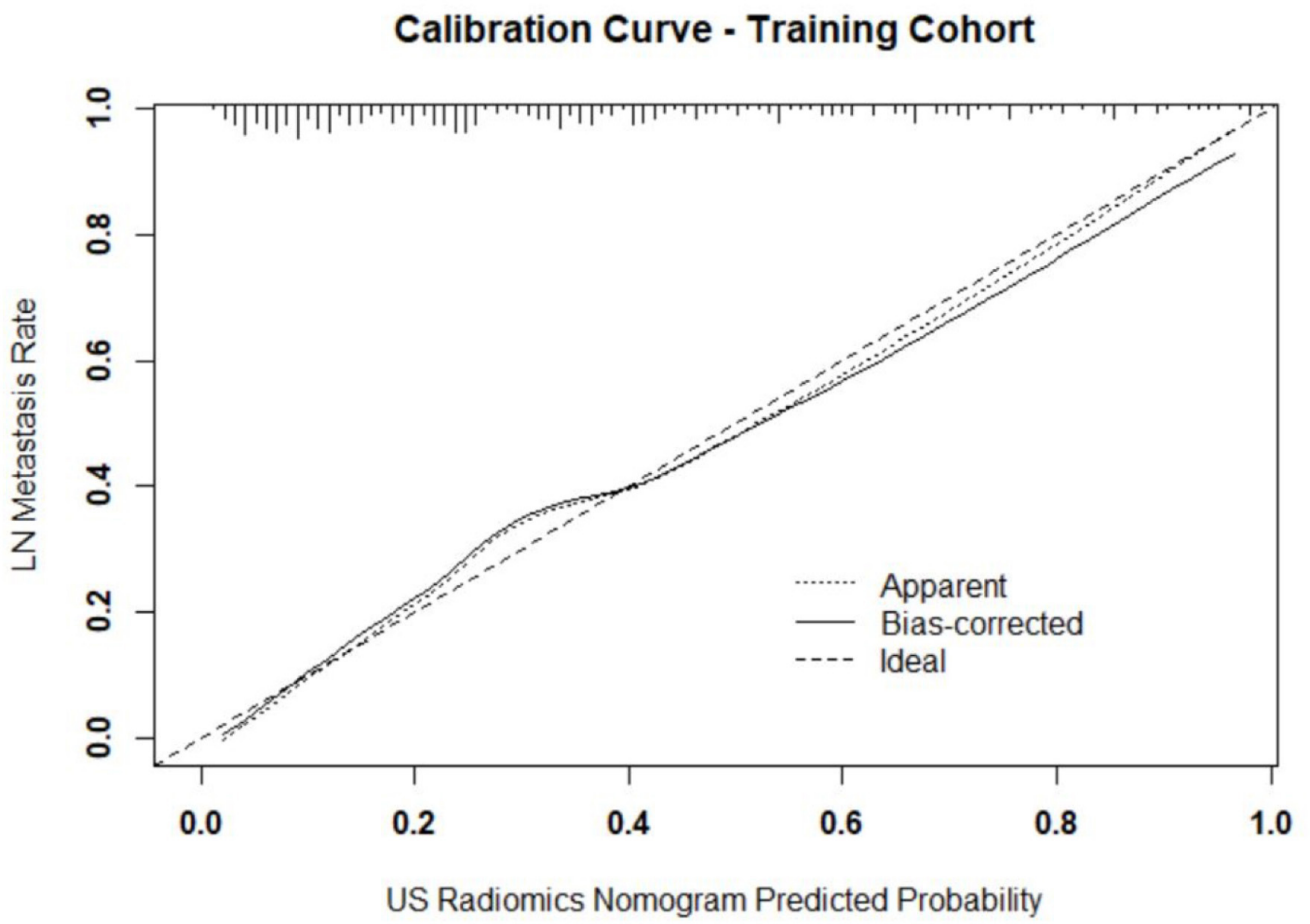
Calibration curve of prediction model in primary cohort. The resultant curve and ideal curve had good consistency, which was confirmed by HL test.

**Figure 4:**
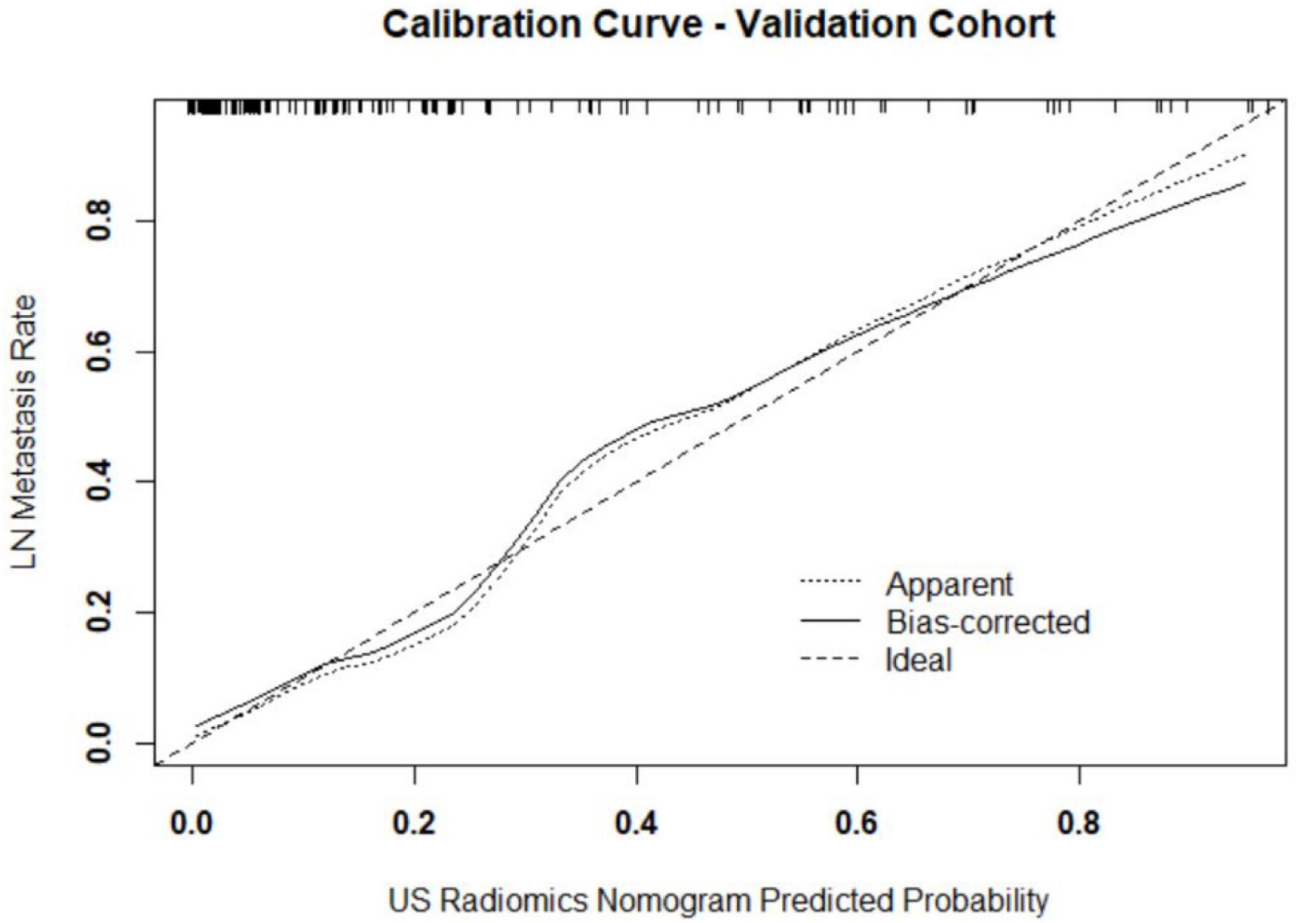
Calibration curve of prediction model in validation cohort. The resultant curve and ideal curve had good consistency, which was confirmed by HL test.

Independent predictors (including age, TG, TPOAB, US radiomics signature, and US-reported LN status) were screened by the logistic regression (Table 4) to establish a nomogram for the prediction of central neck LN metastasis in PTC patients (Figure 2). In the primary cohort, the calibration curve of US radiomics nomogram was delineated for the prediction of central neck LN metastasis and results showed good agreement between prediction curve and standard curve (Figure 3). Hosmer-Lemeshow test showed no statistical significance (*P* = 0.193), which suggests no significant deviation from standard curve. The C-index of nomogram was 0.816 (95% CI, 0.808–0.824) in the primary cohort. In addition, good agreement of calibration curve was also observed in the validation cohort (Figure 4), Hosmer-Lemeshow test showed no statistical significance (*P* = 0.568), and the C-index was 0.858 (95% CI, 0.849–0.867).

**Table 4:** Independent risk factors of US radiomics nomogram after multiple logistic regression analysis.

**Intercept and variable**	**β**	**Odds Ratio (95% CI)**	***P***
Intercept	3.308		0.000
Age	−0.44	0.957 (0.936 to 0.979)	0.000
TG	−1.339	0.017 (0.087 to 0.788)	0.017
TPOAB	−0.712	0.491 (0.270 to 0.892)	0.02
US-reported LN status	1.002	2.724 (1.293 to 5.739)	0.008
US radiomics signature	6.774	874.856 (179.452 to 4265.063)	0.000

*β is the regression coefficient. US radiomics features have a large weight in the prediction model, but the remaining clinical risk indicators also have a considerable contribution*.

### Clinical Significance

**Figure 5:**
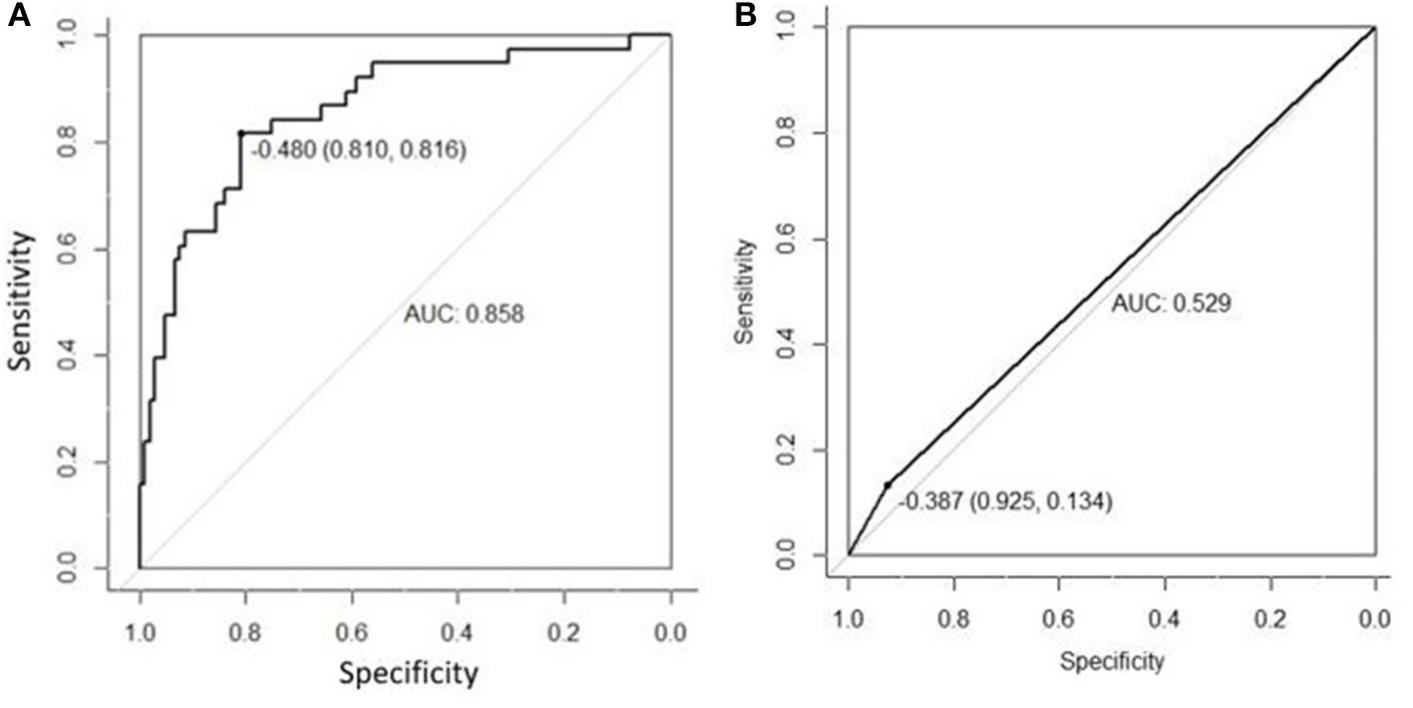
**(A)** ROC curve of prediction model in the validation cohort. **(B)** ROC of pre-operative US in the diagnosis of central cervical LN metastasis of validation cohort. The AUC was significantly different between two cohorts (0.858 vs. 0.529), and the Z test indicated that there was a significant difference in diagnostic performance between two cohorts (*P* = 0.000).

**Figure 6:**
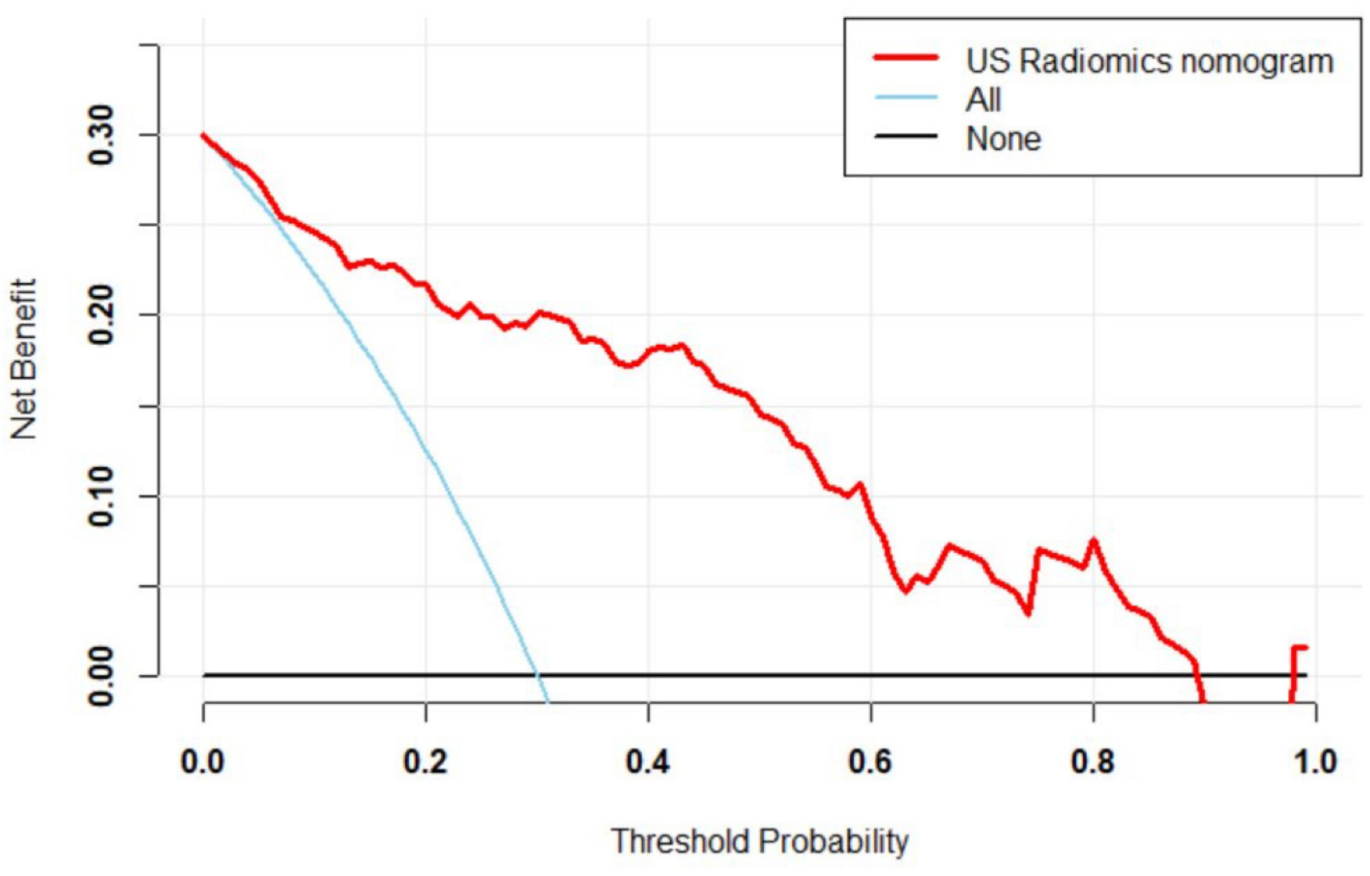
Decision curve of prediction model. Red line: net benefit at different threshold probabilities of US radiomics nomogram. When the probability was 0–0.9, prediction of LN metastasis in PTC patients with US radiomics nomogram could benefit more as compared to all-treated or non-treated patients.

**Figure 7:**
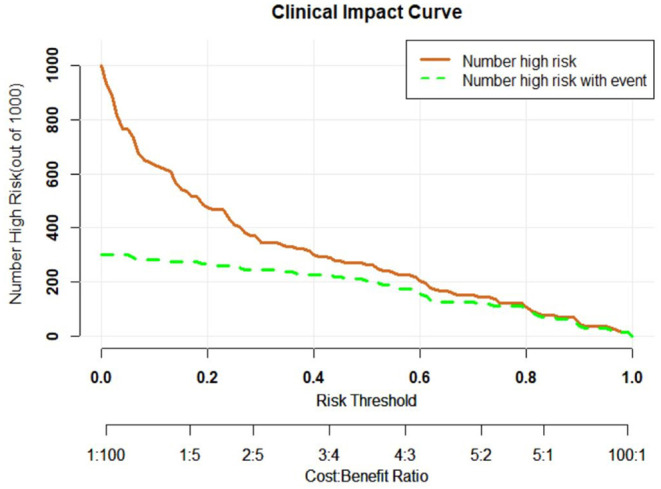
Clinical impact curve of prediction curve. Horizontal ordinate, risk threshold; longitudinal coordinate, positive risk. Yellow solid line, positive rate of prediction model (true positive and false positive values); blue dot line, true positive value. When the risk threshold was 0.4–0.8, the ratio of false positive value to true positive value was lower than 30%.

**Figure 8:**
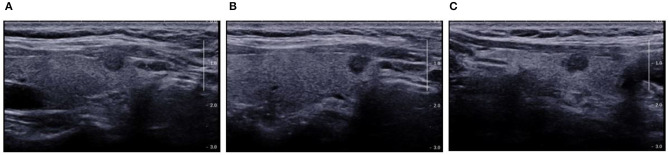
Pre-operative US showed TI-RADS category four and TR score four, and absence of central cervical LN. Cytological pathology showed Bethesda category V. **(A)** Longitudinal section of the lesion. The edges were blurred, some were hard to be differentiated from the thyroid capsule, and there was a trend of outward growth in the thyroid; **(B)** Oblique section of the lesion. The edge was irregular, but there was a distance between the lesion and the capsule; **(C)** Cross-section. The edge was slightly lobular and hard to differentiate from the thyroid capsule, and there was parenchyma hypoechoic inside. Prediction with US radiomics model indicated central cervical LN metastasis. The final pathological examination revealed metastasis in two lymph nodes in the VI area. The radiomics features were independently analyzed and calculated. The scores of radiomics features on the longitudinal section **(A)** and cross section **(C)** were higher (−0.1337 and −0.1390, respectively) than that on the oblique section **(C)** (−0.2288). This suggests the relationship between lesion and thyroid capsule has a high weight in the radiomics features.

**Figure 9:**
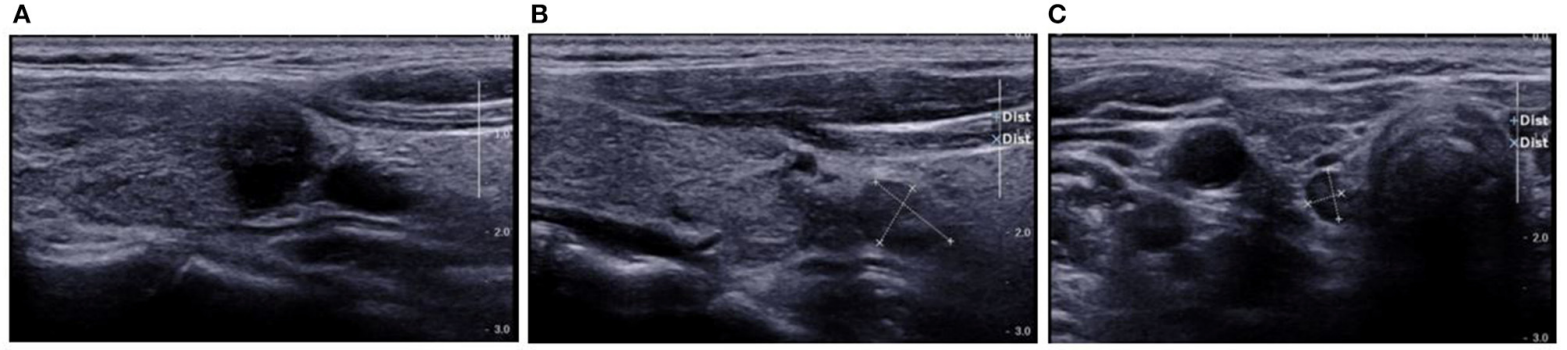
Preoperative US showed TI-RADS category five and TR score eight. Preoperative US showed central cervical LN metastasis. Cytological pathology after FNA showed Bethesda category VI. **(A)** Longitudinal section of the lesion. Evident hypoechoic and blurred edges were observed, the lesion was taller-than-wide and solid-like and hard to be differentiated from the thyroid capsule; **(B)** Longitudinal section of lymph nodes in VI area. The LNs were solid-like and oval, hypoechoic were observed inside the LNs, the lymphatic hilus had ill-defined border and had no calcification; **(C)** Cross-section of the lymph nodes in the contralateral VI area. The features of LNs on US were similar to those of LNs in the affected side. US suggested Hashimoto's thyroiditis, lymph nodes were observed in bilateral VI area, but the lymph nodes of the affected side were larger. Based on the shape, border and echoes of lymphatic hilus, bilateral cervical metastasis was suspected on US. Prediction with US radiomics model showed central cervical LN metastasis, **(A)** the lesion border was spiculated and ill-defined, and hard to differentiate from the thyroid capsule. It was taller-than-wide. It could be identified from the high-weight features on the basis of radiomic features. The final pathological examination showed metastasis in one LN of the VI area, and Hashimoto's thyroiditis was also diagnosed.

In the primary cohort, the accuracy, sensitivity, specificity and AUC were 0.798, 0.825, 0.786, and 0.870 (95% CI, 0.802–0.938), respectively. In the validation cohort, the accuracy, sensitivity, specificity and AUC of this model were 0.812, 0.816, 0.810, and 0.858 (95% CI, 0.785–0.930), respectively. The ROC was plotted (Figure 5A) when US nomogram was used in the validation cohort. In the validation cohort, the accuracy, sensitivity and specificity of pre-operative US in the diagnosis of central cervical LN metastasis were 0.653, 0.134, and 0.925, respectively. Figure 5B is the ROC of pre-operative US, and its AUC was 0.529 (95% CI, 0.493–0.566). Z test showed significant difference between them (*P* = 0.000). The decision curve analysis was used to assess the clinical significance of US radiomics nomogram (Figure 6). Results showed prediction of central neck LN metastasis with US radiomics nomogram could benefit more as compared to all-treated or non-treated patients when the threshold probability ranged from 0 to 0.9. To further evaluate the clinical significance of this prediction model, the clinical impact curve was delineated (Figure 7). When the threshold probability ranged from 0.4 to 0.8, the ratio of false positive value to true positive value, which could be measured on the figure, reduced from 30 to 0 with the increase in the risk. In Figures 8, 9, the pre-operative US images were analyzed combining the results from US radiomics prediction model.

## Discussion

Currently, there is still controversy on prophylactic central neck dissection (35–37). Thus, accurate pre-operative determination of central cervical LN status is clinically important for the selection of therapeutic regiment for PTC. For the pre-operative diagnosis of central cervical LN metastasis, US and CT are the most common tools used currently (12), but their sensitivity is lower than 60% in the diagnosis of central cervical LN metastasis of PTC patients (16–18, 38, 39). The sensitivity of PET/CT is still as low as 48.9% in the diagnosis of PTC (40). Some studies have been conducted to establish the clinical model for the prediction of central cervical LN metastasis in PTC patients (41–44), and their AUC range from 61.5 to 76.4%, which is similar to the diagnosis with combination of US and CT (17, 18). In addition, some investigators have studied the relationship between US image features and central cervical LN metastasis in PTC patients, but prediction model was not established (45, 46). The main reason might be that US is not sensitive and US diagnosis is often subjective. In the machine learning-based radiomics, the image features are extracted via a computer, and self-training and learning are performed based on the pathological results (22). Thus, radiomics provides a chance for the standardized interpretation of US images. Available studies have shown that radiomics based on US or CT images can be used to predict the cervical LN metastasis of PTC (44, 47) with the AUC ranging from 0.727 to 0.795. It has been indicated that the sensitivity and AUC of imaging diagnosis of lateral cervical LN metastasis are higher, which helps to improve the diagnostic accuracy of cervical LN metastasis (16–18, 39). However, the results from the previous studies are not superior to that of central cervical LN metastasis prediction model established with clinical indicators. Therefore, we speculate that imaging examination may not fully reflect the LN metastasis status in the central neck of PTC patients, even machine learning-based radiomics. Thus, in this study, the radiomics features of US were used to generate a score after dimensionality reduction. This score combined with a series of previously reported clinical risk factors was used to construct a model for the prediction of central cervical LN metastases in PTC patients.

Based on previous findings, the extensively studied clinical risk factors (including age, gender, TSH, TG, TGAB, and TPOAB) were investigated in the present study (48–54). Among them, age and TG showed high predictive potential in both univariate (*P* = 0.000 and 0.008, respectively) and multivariate analyses (*P* = 0.000 and 0.017, respectively) and therefore included for the establishment of model. Gender had a good predictive potential in univariate analysis (*P* = 0.008) but showed a poor potential in multivariate analysis (*P* = 0.72) and therefore it was excluded from our model. In addition, TPOAB had a poor predictive potential in univariate analysis, but showed a good potential in multivariate analysis. This might be ascribed to the confounding factors in univariate analysis. Finally, TPOAB was included in the model. Empirically, multifocality is often considered a high-risk factor for the progression of PTC in many studies (55, 56). Meanwhile, multifocal lesion is also an important indication to surgery for PTC patients, and thus it was not included in the prediction model (12). Similarly, ipsilateral or contralateral cervical LN metastasis was not included in the prediction model because they can be easily and accurately identified by US, unlike central neck LN metastasis (57–59). Although preoperative US has a low accuracy in the diagnosis of central cervical LN metastasis, univariate and multivariate analysis in the present study showed US-reported LN status was an independent risk predictor. Considering it is easy to obtain before operation, it was also included in the prediction model. Bethesda category is based on the cytological examination after FNA and can be obtained before surgery. However, multivariate regression analysis showed it was not an independent predictor and thus not included in the prediction mode. This may be explained as that only patients with Bethesda category V or VI were included in the present study. According to the TI-RADS system of ACR, TR score was obtained from each lesion, and this score was based on the macroscopic analysis of this lesion on US image. However, multivariate analysis showed TR score was not an independent predictor of central cervical LN metastasis in PTC patients, which was different from the score of US radiomics features. This indicates that the sensitivity of macroscopic analysis of US images is significantly lower than that of machine-learning based analysis. Finally, TR score was not included in the prediction model.

Whether the US radiomics features are operator-dependent is still unclear in our research. Thus, the inter-operator consistency was further assessed in the extraction of lesion features. The intra-operator correlation coefficient of US doctor-1 in two extractions (0.845–0.962) and the inter-operator correlation coefficient of extraction by US doctor-1 and US doctor-2 (0.886–0.934) were high, suggesting that the extraction of US radiomics features is independent of operator and resolves the operator-dependence in traditional US (60). Furthermore, the stability of extraction of US image features was further assessed in a control cohort, in which a different US machine was used and results were compared between control cohort and validation cohort. The US radiomics features collected from the primary cohort were applied in the validation cohort [images were collected with Supersonic (Acoustics)] and control cohort [images were collected with Voluson E8 (GE)], for the prediction of central cervical LN metastasis in PTC patients. Then, the ROC was delineated and the AUC was calculated (Figure 2). Although the shapes of ROC were different between them, Z test showed no significant difference (0.805 vs. 0.766, *P* = 0.595). Patients in the validation cohort and control cohort were selected from the same period, and the inclusion criteria, exclusion criteria and image requirements were also the same. These findings suggest that this model can be used in different machines as long as the standardized process is used for image acquisition and analysis, which was also consistent with our previous findings on the stability of US radiomics (61).

In the validation cohort, the prediction of central neck LN metastasis in PTC patients with US radiomics nomogram displayed significantly higher accuracy (0.812 vs. 0.653; *P* < 0.01), sensitivity (0.816 vs. 0.134; *P* < 0.01), and AUC (0.858 vs. 0.529; *P* < 0.01) than those of conventional US which was conducted by several US clinicians with more than 10 years' experience in the thyroid ultrasonography. This suggests that the machine-learning based radiomics is superior to experienced clinicians once enough clinical risk information has been provided, which was consistent with previous findings from the artificial intelligence studies on thyroid tumors (62, 63). The predictive efficacy of this model in the validation cohort was compared with previously reported, and results showed the advantage of this model: the sensitivity of this model was 0.816, but that of combined use of CT and US was 0.33–0.66 (15–18); the AUC of this model was 0.858, but the AUC of models established based on different clinical parameters was 0.706–0.764 (41–44). This prediction model containing several risk factors was superior to the model with US radiomics alone (26), demonstrated by both AUC and accuracy. This implies that, although US radiomics had a higher weight in this model, other risk factors were still important for this model (Table 4). All the risk factors in this model can be obtained before surgery, and thus this nomogram can be used for individualized assessment of risk for central cervical LN metastasis in unifocal PTC patients.

In the primary model and validation model, the C-index of this model was good (0.816 vs. 0.858), suggesting that this model has a favorable prediction of LN metastasis; the calibration curve displayed good fitting (Hosmer-Lemeshow test; *P* = 0.193 vs. 0.568) suggesting that this model has good consistency with real condition. Although the ROC curve, C-index and calibration curve can be used to evaluate the predicative value of US radiomics nomogram, it is necessary to further assess the clinical benefit of patients after using this prediction model. Thus, a decision curve was delineated to evaluate the benefit after use of the US radiomics nomogram to predict central neck LN metastasis at different threshold probabilities. Our results showed, when the threshold probability was 0–0.9, patients could benefit more from the prediction of LN metastasis with nomogram. The clinical impact curve showed, when the threshold probability was 0.4–0.8, the ratio of false positive value to true positive value was <30% and decrease to 0 with the increase in the threshold probability. This indicates that the diagnostic accuracy of this model increases and the false positive reduces with the elevation of threshold probability of central cervical LN metastasis, which may avoid unnecessary surgery.

With the introduction of artificial intelligence in recent years, radiomics and deep learning have been widely used in the studies of tumor imaging. Deep learning realizes the end-to-end machine-learning, but the learning process cannot be clearly explained, and the consistency of image data between training and verification cohort should be confirmed. The radiomics based on traditional machine learning requires manual extraction of image features, but the entire process is interpretable, and its features are relatively stable. Currently, this is mainly used the differentiation of malignant tumor from benign tumor in patients with thyroid diseases (47, 62, 64, 65), and little is known about its application in the prediction of LN metastasis in patients with malignant thyroid tumors. Two studies investigated the prediction of whole cervical LN metastasis with radiomics (44, 47), and one investigated the prediction of lateral cervical LN metastasis (25). No study has been conducted to investigate the prediction of central cervical LN metastasis. In the actual clinical situation, the sensitivity of imaging examination can reach 0.7 in the diagnosis of lateral cervical LN metastasis, but it is lower than 0.5 in the diagnosis of central cervical LN metastasis (16, 38, 40, 59, 66). The accurate determination of central cervical LN metastasis directly affects the use of prophylactic LN dissection. Thus, this study focused on the prediction of central cervical LN metastasis in PTC patients. In the US examination, there are often fine adjustments (focus, TGC curve, etc.) even if the image acquisition follows the predesigned requirements, and more interpretable models and processes are often used in clinical practice. Thus, in the present study, machine-learning based radiomics model was used. In the validation cohort, the final AUC (0.858) was the highest in the existing models used for the prediction of LN metastasis; the higher sensitivity meant a more accurate negative prediction rate, which helps reduce unnecessary prophylactic central cervical LN dissection.

**Figure 10:**
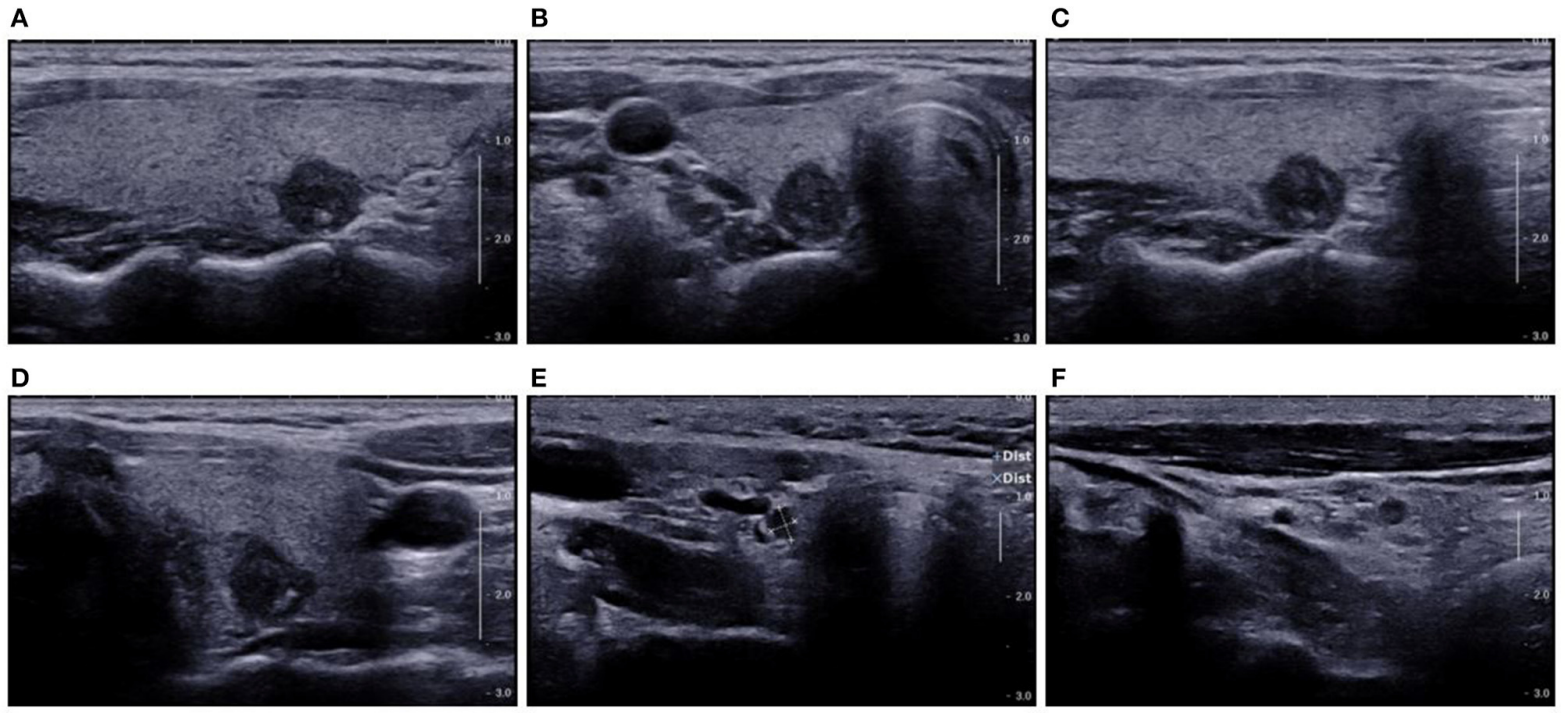
US showed TI-RADS category five and TR score nine. Preoperative US showed potential central cervical LN metastasis. Cytological pathology after FNA showed Bethesda category VI. **(A)** Longitudinal section of the lesion; **(B)** cross-section of the lesion; **(C)** oblique section of the lesion; **(D)** another oblique section of the lesion; **(E)** cross-section of LNs in the VI area; **(F)** Longitudinal section of LNs in the VI area. Prediction with US radiomics model showed central cervical LN metastasis. The final pathological examination indicated no LN metastasis in the VI area. The lesion was spiculated and irregular on the longitudinal section **(A)**, which were not observed in other sections **(B–D)**. This suggests an error in the prediction with US radiomics model. The LNs on the US image were solid-like and round, and had hypoechoic and smooth border, there was loss of echoes of lymphatic hilus, but there was no calcification **(E,F)**. In the absence of Hashimoto's thyroiditis, the LNs were suspected as metastatic LNs according to the loss of echoes of lymphatic hilus and round shape of LNs.

Although the results of this study were promising, there were still some issues that should be interpreted. First, unlike the deep learning process which is performed entirely by the machine itself, the methods for extracting the radiomics features are artificially defined. Although this makes the radiomics interpretable, it also determines that the features in the machine learning process are incomplete. These Incomplete features may cause a certain deviation in the expression of tumor information. This may be the most important problem in the radiomics analysis. Second, we studied misprediction of this model in the validation cohort. We found that some image features that were obvious in the longitudinal planes were not obvious in other planes (Figure 10). Generally, the US clinicians obtain information based one dynamic images for further diagnosis. Thus, a single image may not be representative, which may finally affect the establishment of radiomics model. In a study, deep-learning CT images were used to diagnose the cervical LN metastases in thyroid cancer patients, and the results showed the AUC was as high as 0.953 (66), indicating that multi-sequence CT images may comprehensively reflect the characteristics of tumors and help improve the diagnostic efficiency. This also provides a reference for US radiomics: we can acquire more lesion information from more US images. How to formulate standardized multi-cutting planes should be further studies, which is different from CT. Third, the suspected cervical LNs should be subjected to FNA before surgery. When there is conflict between US examination and model prediction, clinical decision making will be difficult. It is possible that US can't identify the metastatic LN when the prediction with this model shows positive metastasis, or ultrasound identify several LNs. This will be a challenge in the clinical application of this model, and more prospective multicenter studies are needed to validate the value of this model.

There were several limitations in this study. First, there were only 609 patients in the primary cohort and 326 patients in the validation cohort. The sample size was still not large enough for the analysis of US radiomics features. Second, the gene mutation was not included in our study. In recent years, increasing studies have been conducted to investigate the gene radiomics and results reveal that gene mutation is related to LN metastasis in PTC (49, 67–69). This study was a retrospective study, and not all the patients received BRAF examination after pre-operative FNA. Thus, the role of gene mutation as an independent predictor is needed to be further studied. Third, although the stability of US radiomics features was confirmed in our study, validation is needed in more multicenter studies with different US machines for image acquisition. To solve these problems, a multicenter study with large sample size is ongoing, in which the US radiomics features collected from different US instruments and by different operators were analyzed, and we are expecting promising findings.

## Conclusion

In conclusion, a prediction model is established based on US radiomics signature and clinical risk factors, and it is convenient to assist clinician in individually predicting central neck LN metastasis of PTC patients.

## Data Availability Statement

The datasets generated for this study are available on request to the corresponding author.

## Ethics Statement

The studies involving human participants were reviewed and approved by the Ethics Committee of Cancer Hospital of Fudan University. The patients/participants provided their written informed consent to participate in this study.

## Author Contributions

S-CZ, T-TL, JZ, and CC designed this study. S-CZ, T-TL, JZ, Y-XH, YG, J-HY, and Y-YW conducted the experiment and interpreted the data. S-CZ, T-TL, and JZ analyzed the data. All authors approved the final version of this manuscript.

## Conflict of Interest

The authors declare that the research was conducted in the absence of any commercial or financial relationships that could be construed as a potential conflict of interest.
